# Age-Related Difference in Cognitive Performance under Severe Whole-Body Hyperthermia Parallels Cortisol and Physical Strain Responses

**DOI:** 10.3390/medicina59091665

**Published:** 2023-09-14

**Authors:** Junli Wang, Rima Solianik, Nerijus Eimantas, Neringa Baranauskiene, Marius Brazaitis

**Affiliations:** Institute of Sport Science and Innovations, Lithuanian Sports University, LT-44221 Kaunas, Lithuania; junwan@stud.lsu.lt (J.W.); rima.solianik@lsu.lt (R.S.); nerijus.eimantas@lsu.lt (N.E.); neringa.baranauskiene@lsu.lt (N.B.)

**Keywords:** hyperthermia, heat stress, cognitive functioning, thermoregulation, aging

## Abstract

*Background and Objectives*: To date, understanding age-related changes in cognitive processes during heat exposure still needs to be better-understood. Thus, the main aim of the current study was to evaluate the effects of whole-body hyperthermia (WBH), i.e., a ≈ 2.5 °C increase in rectal temperature (Tre) from overnight-fast baseline value, on cognitive functioning in old and young men and to explore factors, such as stress and thermophysiological strain, that could influence such changes. *Materials and Methods*: Ten young (19–21 years of age) and nine old (61–80 years of age) healthy men underwent an experimental trial with passive lower-body heating in hot water immersion (HWI) at 43 °C (HWI–43 °C) until Tre reached 39 °C in old adults and 39.5 °C in young adults. Cognitive performance and cortisol concentration were assessed before and after HWI, and the physiological strain index (PSI) was assessed during HWI–43 °C. *Results*: PSI was lower and cortisol concentration was greater after HWI–43 °C in the old group compared with the young group (*p* < 0.05). Surprisingly, hyperthermia improved cognitive flexibility only in old adults, whereas short-term and visual recognition memories were maintained in both age groups. *Conclusions*: A ≈ 2.5 °C increase in rectal temperature can improve executive function in old adults, and this increase parallels the increased cortisol concentration and the lower thermophysiological strain under severe WBH conditions.

## 1. Introduction

More than one-fifth (21.1%) of the European Union (EU) population was aged 65 years and over in 2022 [1], and in the EU and worldwide this population is expected to increase rapidly in the coming years [2]. In parallel, heat waves have increased in intensity, frequency, and duration, and heat wave changes are projected to worsen under enhanced global warming [3]. More frequent and intense periods of extreme heat are threatening human health, and old adults are particularly vulnerable [4]. Moreover, the elevated environmental temperature could cause a cognitive deficit, thus bringing about a reduction in working capacity, productivity, and safety. As a result, studies of cognitive performance under heat exposure are of great importance [5].

The aging process is linked to structural and functional changes in the brain, leading to a decline in cognitive performance. The prefrontal cortex (PFC) and hippocampus volume decrease by 1–2% annually in individuals over 55 years of age [6]. Changes in brain volume, especially in prefrontal regions and the hippocampus, have been suggested to account for the age-related cognitive decline often occurring in memory and executive functions tasks [7,8]. Additionally, separate brain regions that interact to subserve higher-order cognitive functions show less-coordinated brain activation with aging; this reduced coordination between the anterior medial PFC with posterior cingulate/retrosplenial is associated with poorer cognitive functioning, including executive functioning [9]. Paradoxically, previous studies with exogenous administration of glucocorticoids have established that the PFC and hippocampus are particularly sensitive to stress, and PFC-related cognitive functions are more sensitive to acute stress than hippocampus-related cognitive functions [10,11]. Therefore, age-related cognitive decline could be further exacerbated by stress, putting old adults at a greater risk of cognitive-functioning accidents at work or in daily life.

The severity of heat stress is directly proportional to the rise in core body temperature [12,13,14]. When the core body temperature reaches 38.3 °C and more, it can result in whole-body hyperthermia (WBH) [15]. Liu et al. (2013) established that passive hyperthermia impaired executive function [16], especially efficient conflict resolution. Even prehyperthermic core temperature increases to 37.9 ± 0.3 °C induce executive control changes, accompanied by altered functional connectivities. Decreased connectivities were mainly focused on the PFC, temporal lobe, and occipital lobe, and increased connectivities were mainly located within the limbic system. The greater the number of hyperthermia-induced altered functional connectivities, the greater the executive control decline evoked [17]. In contrast to specific brain areas’ sensitivity to stress, it was established that WBH (rectal temperature (Tre) = 39.5 °C) in young men does not affect hippocampus-related visual recognition memory or PFC-related cognitive flexibility and short-term working memory [18,19,20]. It was suggested that the negative effects of passive hyperthermia on cognitive processes could be overcome through variant regional brain activation. For example, functional magnetic resonance imaging showed that (1) WBH increased the activity in the right superior frontal gyrus and decreased the activity in the right middle occipital gyrus, left inferior parietal lobule, and left culmen in the alerting network; and (2) passive hyperthermia increased the activity in the temporal lobe and decreased the activity in the frontal lobe, parietal lobe, and occipital lobe in the orienting network [16].

It was established that similar cognitive task performance responses for young and old adults accompany a mild Tcore increase of 1 °C [21]. However, a recent study established that despite lower thermophysiological strain (i.e., lower heart rate and lower thermal sensation), a core temperature increase of ≈2.5 °C is accompanied by greater cortisol response in old men than young men [22]. Thus, in this case, two alternative hypotheses could be expected. First, it can be expected that an increase in cortisol would decrease cognition, specifically PFC-related functioning. Second, it can be expected that WBH-induced lower thermophysiological strain in old adults would compensate for this decline in cognitive functioning. Therefore, the current study aimed to evaluate the effects of severe WBH (a Tre increase of ≈2.5 °C) on cognitive functioning in old and young men and to explore factors, such as stress and thermophysiological strain, that could influence such changes. In addition, different brain-area-related cognitive tasks (i.e., hippocampus-related visual recognition memory, PFC-related cognitive flexibility, and short-term working memory) [19,20] were investigated to evaluate if WBH evokes brain-area-specific responses.

## 2. Materials and Methods

### 2.1. Participants

The number of participants was selected based on the calculated sample effect size (G*Power for Windows; version 3.1.9.4; Düsseldorf, Germany), following the use of the data involving three young and three old healthy subjects who completed the study. At an α of 0.05 and β (*x*) value of 80%, our power analysis indicated that six participants per group would be required to detect a main large effect of 2.0 for hypothesized executive-functioning-related parameters (change from baseline of cognitive flexibility and working memory) (*n* = 12 in total). Furthermore, six participants (three young and three old adults) were added after considering the attrition rate and missing data.

Advertisements through public seminars and social networks were used for participant recruitment. The criteria for inclusion were: (1) young (18–23 years of age) or old (65–81 years of age) adult men; (2) not participating in any formal physical exercise or sports program; (3) no involvement in any temperature-manipulation program or extreme temperature exposure in the previous three months; (4) nonsmokers; and (5) no diseases, conditions, or medications that could affect natural thermoregulation or cognition and that could be worsened by exposure to hot water. Eleven young (19–21 years) and nine old (61–80 years) healthy male subjects were initially recruited for the current study. One young participant did not provide data on cognitive functioning and therefore was excluded from the study. Thus, the final study sample comprised 19 participants. The baseline characteristics of the participants are presented in Table 1. Old men had a higher body fat percentage (Mann–Whitney *U* = 10.00, *p* = 0.003), whereas anthropometric characteristics did not differ between young and old men.

### 2.2. Experimental Procedures

**Figure 1:**
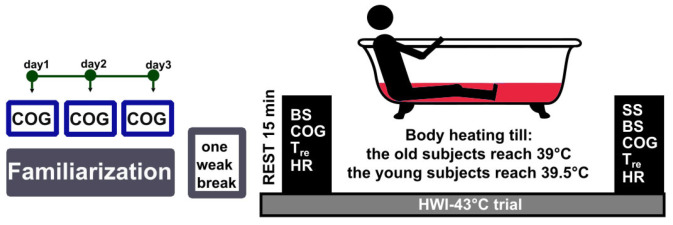
Schematic representation of the protocol. Notes. HWI–43 °C = passive lower-body heating in 43 °C water, BS = blood sampling, COG = cognitive functioning, Tre = rectal temperature, HR = heart rate, SS = subjective sensations.

The study comprised two trials with the same participants, a familiarization trial and an experimental trial (Figure 1). To ensure consistency in performance, participants were introduced to the experimental procedures and asked to practice the cognitive testing exercises until a stable level of performance was achieved one week before the experiment. The experiment was conducted around 8:00 am on the trial day to avoid circadian rhythms’ influence. To standardize the hydration status of the participants, they were allowed to drink still water as per their requirement until 60 min before the experiment. The study was conducted in a room maintained at a temperature of 24 °C and relative humidity of 60%.

All experiments were conducted at a consistent time of day to prevent any potential variations in thermoregulatory and hormonal responses due to circadian rhythms [23,24]. The participants received instructions to sleep for at least 7–8 h the night before the experiment. They were also advised to avoid consuming alcohol, engaging in heavy exercise, or consuming caffeine for at least 24 h before the experiment. In addition, they were instructed to abstain from consuming food for at least 12 h before arriving at the laboratory.

### 2.3. Study Design and Experimental Protocol

A before and after study design was used (Figure 1). The study occurred at the Institute of Sports Science and Innovations, Lithuanian Sports University, from January 2015 to October 2017. Upon arriving at the laboratory, body weight was measured, a rectal thermometer was inserted to record the internal temperature, and a heart rate (HR) sensor was attached to the chest. To ensure a stable condition, the subjects were instructed to rest semi-recumbently for 15 min while wearing a T-shirt, swim shorts, and socks. HR (see Section 2.4.3) was recorded, and a blood sample (see Section 2.4.7) was taken upon reaching a rest of 15 min. Within 2 min of these resting measurements, the participant was seated at a table and underwent cognitive function testing (see Section 2.4.6). Then, the participants were immersed up to the waistline in a water bath with a temperature of approximately 43 °C. HR and Tre (see Section 2.4.2) were recorded throughout immersion. Several epidemiological studies have shown that geriatric men (aged 65–95 years) generally have a lower internal (core) body temperature (by approximately 0.4 °C) than younger adult men (aged 20–64 years) [14]. Given this, the immersion was continued until the participant’s Tre increased by approximately 2.5 °C; at this point, the immersion ceased for the young group at a Tre of 39.5 °C and for the old group at a Tre of 39.0 °C. The duration of the exposure required to achieve this Tre was recorded. Once the target Tre was reached, ratings of subjective sensations (see Section 2.4.5) were recorded. Within one minute of exiting the water bath, the participants were towel-dried, their cognitive function testing was repeated, and their body weight was assessed.

### 2.4. Measures

#### 2.4.1. Measurement of Sweating Rate

The participant’s body weight was measured using a Tanita TBF-300 body composition analyzer (West Drayton, UK), and the sweating rate was assessed using the following equation: sweating rate = body weight before HWI–43 °C − body weight after HWI–43 °C.

#### 2.4.2. Measurement of Rectal Temperature

The participant’s Tre was measured at rest and throughout the immersion procedure using a thermocouple (Rectal Probe; Ellab, Hvidovre, Denmark; accuracy ±0.01 °C) inserted to a depth of 12 cm beyond the anal sphincter. Each participant placed the rectal thermistor sensor.

#### 2.4.3. Measurement of Heart Rate

The participant’s HR was measured at rest and immersion using a Polar HR monitor (RCX5; Kempele, Finland). The HR data were downloaded to a computer equipped with the Polar Pro Trainer version 5 software (Polar, Vantaa, Finland), where data were analyzed.

#### 2.4.4. Measurement of Physiological Strain Index

The approach utilized to measure the physiological strain index (PSI) has been described in previous research [25]. The normalized PSI was computed using the following formula: PSI = 5 (Tret − Tre0) × (39.0 [or 39.5] − Tre0)^−1^ + 5 (HRt − HR0) × (180 − HR0)^−1^. Measurements for PSI were collected prior to passive heating (Tre0 and HR0) and at the conclusion of passive heating (Tret and HRt). Tre and HR were given equal weight by a constant value of 5. The index was scaled to a range of 0–10: 1–2 (no or minimal heat stress), 3–4 (low heat stress), 5–6 (moderate heat stress), 7–8 (high heat stress), and 9–10 (very high heat stress), with the following limits: 36.5 ≤ Tre ≤ (end Tre of 39.0 °C for old men) or (end Tre 39.5 °C for young men) and 60 ≤ HR ≤ 180 bpm.

#### 2.4.5. Measurement of Subjective Sensations

The scales used to evaluate the subjective thermal, sweating, and comfort sensations for the entire body have been described previously [18,22], and these scales are presented in Table 2. Participants provided their subjective sensations when their Tre reached 39 °C for old individuals and 39.5 °C for young individuals.

#### 2.4.6. Measurement of Cognitive Functioning

A programmed cognitive test battery assessed cognitive functioning before and after HWI–43 °C. All tasks were computer-controlled, and the information was presented on the screen of a laptop (Samsung R538 (San Jose, CA, USA)) positioned 40 cm in front of the participant. Measurement of cognitive functioning was performed in a quiet and semi-darkened laboratory. The cognitive test battery included an odd/even test to evaluate cognitive flexibility, a forward digit-span test to evaluate short-term working memory, and a forced-choice recognition memory test to assess visual recognition memory [18,26,27,28]. The reliability of the chosen tests was considered acceptable, as indicated by the intraclass correlations of *R* ≥ 0.80 and a coefficient of variation for repeated tests of less than 5% [18,27]. The cognitive test battery took ≈10 min to perform and included the tests presented in randomized order.

##### Forced-Choice Recognition Memory Test

After viewing 9 visual figures presented for 15 s in the center of the screen, the participant was instructed to recognize these figures from a list of 28 in any order. The number of correctly identified images was recorded.

##### Forward Digit-Span

The participant was instructed to remember a seven-digit sequence displayed for three seconds in the center of the screen. The participant then immediately entered the seven-digit sequence using a numeric keyboard in the same consecutive sequence as presented. If the digits were identified correctly, for the next attempt, the sequence was increased by one digit; if the digits were identified incorrectly, the next sequence was decreased by one digit. There were 16 sequences, and the mean number of digits identified correctly was recorded.

##### Odd/Even Test

The test comprised 40 randomized single-digit stimuli ranging from 0 to 9; each was presented for 180 s with varying interstimulus intervals in the center of the screen. The participant was instructed to press the appropriate button corresponding to the digit presented (right for even, left for odd) as quickly as possible.

#### 2.4.7. Measurement of Cortisol Concentration

Venous blood samples were collected via venipuncture into vacuum tubes for a serum containing a gel separator (5 mL) before and after HWI–43 °C. Blood samples were allowed to clot for 30 min, and serum was separated via centrifugation (1200× *g*, 15 min) at 4 °C. Then, the separated serum samples were stored at −80 °C until analysis. The cortisol concentration was measured using an AIA-2000 automated enzyme immunoassay analyzer (Tosoh Corp, Tokyo, Japan).

### 2.5. Data Analyses

Statistical analysis was performed using IBM SPSS Statistics for Windows (version 28.01.0; Armonk, NY, USA). Data are reported as mean (M) ± standard deviation (SD). Because of the small sample size, the Mann–Whitney nonparametric *U* test was used to compare results between the young and old groups. The Wilcoxon signed-rank test was used to compare the results within the experimental trial (before and after HWI–43 °C). The level of significance was set at *p* ≤ 0.05. If a significant effect was found, the effect size (*r*) was calculated by dividing the absolute standardized test statistic *Z* by the root mean square of the number of pairs (for the Wilcoxon signed-rank test) or by the root mean square of the sample size (for the Mann–Whitney *U* test). The commonly accepted interpretation of *r* values was used: small effect size: *r* = 0–0.3, moderate effect size: *r* = 0.3–0.5, and large effect size: *r* ≥ 0.5.

## 3. Results

### 3.1. Effects of HWI on Sweating Rate and Subjective Sensations

Old men tended to feel cooler (Mann–Whitney *U* = 25.00, *p* = 0.054, *r* = 0.44); however, sweating and comfort sensation did not differ between young and old men (Table 3). The mean sweating rate adjusted to time spent in the bath in old men was lower (0.015 (0.001) vs. 0.025 (0.002) g min^−1^; Mann–Whitney *U* = 13.00, *p* = 0.012, *r* = 0.65) than that in young adult men.

### 3.2. Physiological Response to Passive Heating

The physiological response to passive heating is presented in Table 4. Baseline HR did not differ between young and old men. The Wilcoxon signed-rank test showed that passive heating increased HR (*Z* = −2.67, *p* = 0.008, *r* = 0.89 for the old group; *Z* = −2.80, *p* = 0.005, *r* = 0.89 for the young group) in both groups. A significantly lower HR after HWI–43 °C was observed in the old group than in the young group (Mann–Whitney *U* = 9.00, *p* = 0.002, *r* = 0.69), and HR significantly increased more in the young group (81.73 (20.91) bpm) compared with the old (56.44 (9.99) bpm) group (Mann–Whitney *U* = 8.50, *p* = 0.003, *r* = 0.68). Furthermore, old men’s HR during HWI–43 °C was lower than young participants (Mann–Whitney *U* = 11.00, *p* = 0.003, *r* = 0.67). The heating duration required for old men to reach the required Tre was longer than that for young men (Mann–Whitney *U* = 15.50, *p* = 0.016, *r* = 0.55), and their PSI (Mann–Whitney *U* = 9.00, *p* = 0.003, *r* = 0.67) was lower than that of young participants.

**Figure 2:**
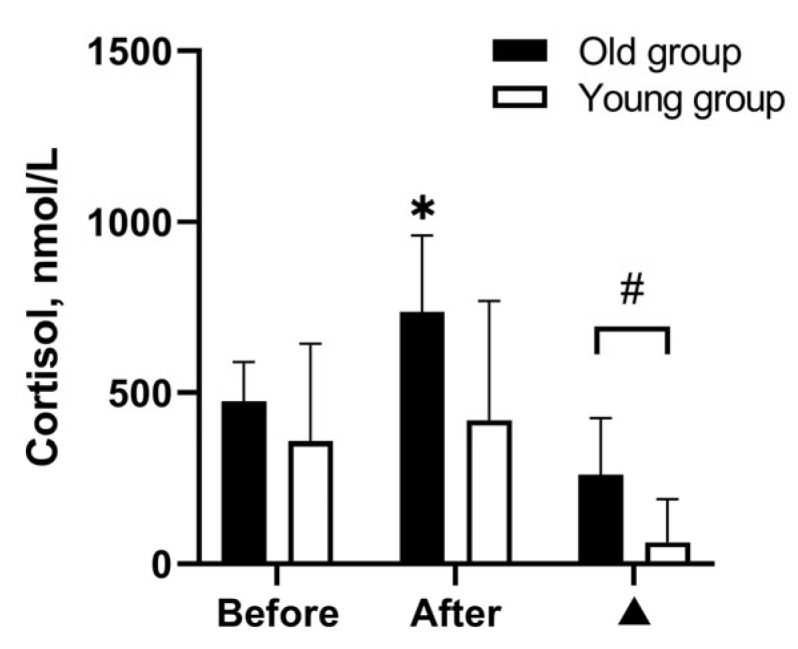
Effects of severe whole-body hyperthermia on cortisol concentrations of old (*n* = 9) and young men (*n* = 10). Notes. Data are presented as mean (standard deviation). ▲ = change from baseline. * *p* < 0.05, compared with before passive lower-body heating in 43 °C water; # *p* < 0.05, compared with old group.

The cortisol response to passive heating is presented in Figure 2. Baseline cortisol concentration did not differ between young and old men. The Wilcoxon signed-rank test showed that WBH increased cortisol concentration only in the old group (*Z* = −2.67, *p* = 0.008, *r* = 0.89), and cortisol change was significantly greater in the old group than in the young group (Mann–Whitney *U* = 14.00, *p* = 0.019, *r* = 0.55).

### 3.3. Effects of WBH on Cognitive Functioning

The cognitive functioning response to passive heating is presented in Table 5. Before WBH, young adults identified a greater number of figures during the forced-choice recognition memory test than old adults (Mann–Whitney *U* = 6.00, *p* < 0.001, *r* = 1.10), and this difference was maintained after WBH (Mann–Whitney *U* = 8.00, *p* = 0.002, *r* = 1.02). The Wilcoxon signed-rank test showed that WBH improved reaction time during odd/even tests only in old adults (*Z* = −2.31, *p* = 0.021, *r* = 0.77). Nevertheless, young adults had significantly faster reaction times after HWI–43 °C (Mann–Whitney *U* = 17.00, *p* = 0.022, *r* = 0.52), and a similar tendency was also observed before HWI–43 °C (Mann–Whitney *U* = 22.00, *p* = 0.060, *r* = 0.43).

## 4. Discussion

We investigated the effect of WBH on cognitive performance in healthy old and young men, with an increase in rectal temperature of ≈2.5 °C (ΔTre 2.5 °C) from overnight-fast baseline value. Our main finding was that healthy elderly individuals improved cognitive flexibility with an increase in Tre of ≈2.5 °C, but their memory was maintained. This improvement paralleled the lower physiological strain and greater cortisol response to WBH compared with young adults.

Regarding cognitive functioning, we found that visual working memory and executive functioning were worse in the older adults than in their younger counterparts. Our data suggested that attenuated cognitive functioning is linked with advanced age, consistent with previous research [6,29,30,31,32]. Surprisingly, WBH improved cognitive flexibility only in the old adult group. The PFC is responsible for executive functioning, and one of the core executive functions is cognitive flexibility [20]. It has been suggested that PFC is the brain region most sensitive to the effects of stress exposure [11], and an inverted U-shape function between stress hormones and cognitive processes has been reported in previous human and animal studies, as very low or high levels of stress hormones impair it, whereas moderate levels can improve it [10,11,33]. Therefore, WBH-induced cortisol increases together with lower thermal distraction in old adults supports the improvement of PFC-related cognitive flexibility. By contrast, PFC-related working memory was not affected. These results support the previous findings that cortisol can modulate cognitive processes and that the effects of cortisol on executive functions are selective [34].

It is worth noting that the chosen cognitive tasks had motor elements (i.e., tests were performed with the dominant right hand); thus, spinal- and supraspinal-level changes can affect task performance. In general, older individuals have longer latencies, smaller amplitudes, and slower conduction velocities than younger individuals [35,36]. Previously, it was established that WBH accelerates spinal and supraspinal excitability in the transmission of neural drive, along with greater response in old adults than in young adults [35]. Consequently, WBH supports improved reaction times in old adults and maintains cognitive processes in young adults.

Young adults are worse than old adults at ignoring distracting negative stimuli [37]. Nevertheless, young adults spent a shorter time in HWI–43 °C; young adults tended to show greater thermal perception of heat (see Table 3) and greater physical strain (PSI; see Table 4) than old adults, which is in line with recent studies [22,38]. Therefore, one plausible explanation for the limited improvements in cognitive flexibility in young men is the distraction hypothesis. Based on this hypothesis, it can be assumed that greater physical and psycho-emotional strain provide competing stimuli that interfere with the response elicited by the reaction signal, and thus produce increased latencies [39].

### Limitations and Future Directions

Our study has several limitations. First, our experiment included only nine old and ten young subjects. Nevertheless, significant changes had large effects (*r* = 0.55–1.10) within and between groups. Second, the generalizability of the results is limited because the sample included only healthy individuals. Thus, it would be beneficial to replicate this research in old individuals with cognitive impairments and to determine whether any cognitive improvements may occur in such groups. Furthermore, individuals with obesity, hypertension, pulmonary or cardiovascular disease, or long-standing diabetes have physiological impairments in regulating body core temperature in hot conditions [40], and the physiological and cognitive responses to passive heating may also differ. Thus, this aspect should be addressed in future research. Third, acute short-term WBH was induced in young and old men. Therefore, the results of WBH should be interpreted cautiously when extrapolating for prolonged WBH (e.g., climatic heat wave conditions). Fourth, we cannot separate and evaluate the degree of influence of the effects of increased cortisol and decreased latency at the spinal and supraspinal level on improved reaction time in old adults, and further mediation analysis with a greater sample size is required to evaluate whether WBH-induced changes in serum cortisol and latency mediate the improvement in cognitive flexibility. Fifth, the importance of further studies to examine the association between age-related emotional regulation differences and WBH-induced evoked cognitive improvement should be pointed out. As discussed earlier, older adults are better than young adults at ignoring distracting negative stimuli [37], which may favor cognitive improvements. Sixth, acute short-term thermal stimuli may evoke a residual response to cortisol [41]. Still, a recent study did not evaluate the residual response to cortisol and its effect on cognitive function. Therefore, evaluating if the evoked cognitive improvement is maintained in old adults is important. Lastly, only men were included in the current study, and the results cannot be applied to women. It has been well-demonstrated that core body temperature changes across the menstrual cycle in women [42] and is lower in postmenopausal women than premenopausal women [43]. Therefore, the response to WBH might differ between cycles, between men and women, and might show age-related differences between women. Future studies should consider including women to gain a more comprehensive understanding of the gender-related similarities and differences in responses.

## 5. Conclusions

Our data showed that a ≈ 2.5 °C increase in rectal temperature greatly affects executive function improvement in old adults, and this increase may be related to increased cortisol concentration and lower thermophysiological strain under severe WBH conditions.

## Figures and Tables

**Table 1:** Characteristics of the participants (*n* = 19).

Measure	Young Men (*n* = 10) M (SD)	Old Men (*n* = 9) M (SD)	*p*
Age, years	20.8 (0.6)	68.9 (5.7)	<0.001
Height, cm	180.8 (6.5)	176.6 (6.4)	0.182
Weight, kg	78.3 (11.1)	83.3 (15.7)	0.494
Body mass index, kg/m^2^	23.9 (3.0)	26.5 (3.3)	0.149
Body fat, %	15.6 (6.13)	24.9 (6.7)	0.003
Body surface area, m^2^	1.98 (0.15)	2.00 (0.21)	0.849

Notes. M = mean; SD = standard deviation; *n* = participant’s number.

**Table 2:** Measurement of subjective sensations.

Rating	Thermal Sensation	Shivering/Sweating Sensation	Comfort Sensation
1	Very cold	Vigorously shivering	Comfortable
2	Cold	Moderately shivering	Uncomfortable
3	Cool	Slightly shivering	Little comfortable
4	Slightly cool	Not at all	Comfortable
5	Neutral	Slightly sweating	Extremely uncomfortable
6	Slightly warm	Moderately sweating	
7	Heavily sweating	Sweating running off in many places	
8	Hot		
9	Very hot		

**Table 3:** Sweating rate and subjective response to passive heating in old and young men (*n* = 19).

Measure	Old Group (*n* = 9) M (SD)	Young Group (*n* = 10) M (SD)
Weight loss, kg	1.36 (0.34)	1.43 (0.38)
Comfort sensation, points	1.56 (0.73)	2.10 (0.74)
Sweating sensation, points	5.67 (0.50)	5.85 (0.47)
Thermal sensation, points	7.00 (0.50)	7.50 (0.53)

Notes. M = mean; SD = standard deviation.

**Table 4:** Physiological response to passive heating in old and young men (*n* = 19).

Measure	Group (*n*)	Before HWI–43 °C M (SD)	After HWI–43 °C M (SD)
HR, bpm	Old (9)	58.22 (10.07)	114.67 (12.32) *
	Young (10)	66.7 (12.34)	149.3 (24.71) *,#
HR during HWI–43 °C, bpm	Old (9)	96.8 (12.0)	
	Young (10)	134.1 (21.1) #	
PSI during HWI–43 °C	Old (9)	7.32 (0.41)	
	Young (10)	8.68 (0.97) #	
Time to target Tre, min	Old (9)	88.2 (16.2)	
	Young (10)	62.7 (21.3) #	

Notes. M = mean; SD = standard deviation; *n* = participant’s number; HWI–43 °C = passive lower-body heating in 43 °C water; HR = heart rate; PSI = physiological strain index. * *p* < 0.05, compared with before HWI–43 °C; # *p* < 0.05, compared with the old group.

**Table 5:** Effects of WBH on cognitive functioning of old and young men.

Measure	Group (*n*)	Before HWI–43 °C M (SD)	After HWI–43 °C M (SD)	▲ M (SD)
Forced-choice recognition memory test				
Figures identified, *n*	Old (9)	4.89 (0.93)	5.11 (1.27)	0.22 (1.20)
	Young (10)	7.30 (1.06) #	7.70 (1.49) #	0.40 (1.90)
Forward digit-span test				
Mean digits identified, *n*	Old (9)	6.35 (0.67)	6.39 (0.65)	0.04 (0.57)
	Young (10)	6.65 (0.58)	6.45 (0.63)	−0.21 (0.40)
Odd/even test				
Reaction time, ms	Old (9)	692.4 (105.0)	636.5 (49.2) *	−55.8 (61.5)
	Young (10)	600.5 (83.4)	587.5 (82.3) #	−13.0 (55.0)

Notes. M = mean; SD = standard deviation; ▲ = change from baseline; *n* = participant’s number; HWI–43 °C = passive lower-body heating in 43 °C water. * *p* < 0.05, compared with before HWI–43 °C; # *p* < 0.05, compared with the old group.

## Data Availability

The findings of this study are supported by data that can be obtained upon request from Marius Brazaitis.

## References

[1] Eurostat Population Structure and Ageing. https://ec.europa.eu/eurostat/statistics-explained/index.php?title=Population_structure_and_ageing.

[2] Shimizu M., Kobayashi T., Chiba H., Senoo I., Ito H., Matsukura K., Saito S. (2020). Adult Spinal Deformity and Its Relationship with Height Loss: A 34-Year Longitudinal Cohort Study. BMC Musculoskelet. Disord..

[3] Perkins-Kirkpatrick S.E., Lewis S.C. (2020). Increasing Trends in Regional Heatwaves. Nat. Commun..

[4] Meade R.D., Akerman A.P., Notley S.R., McGinn R., Poirier P., Gosselin P., Kenny G.P. (2020). Physiological Factors Characterizing Heat-Vulnerable Older Adults: A Narrative Review. Environ. Int..

[5] Zhu H., Hu S., Yu C.W. (2022). Cognitive Performance in a Warming Planet. Indoor Buil. Environ..

[6] Raz N., Lindenberger U., Rodrigue K.M., Kennedy K.M., Head D., Williamson A., Dahle C., Gerstorf D., Acker J.D. (2005). Regional Brain Changes in Aging Healthy Adults: General Trends, Individual Differences and Modifiers. Cereb. Cortex..

[7] Reuter-Lorenz P.A., Park D.C. (2010). Human Neuroscience and the Aging Mind: A New Look at Old Problems. J. Gerontol. B Psychol. Sci. Soc. Sci..

[8] Bherer L. (2015). Cognitive Plasticity in Older Adults: Effects of Cognitive Training and Physical Exercise. Ann. N. Y. Acad. Sci..

[9] Andrews-Hanna J.R., Snyder A.Z., Vincent J.L., Lustig C., Head D., Raichle M.E., Buckner R.L. (2007). Disruption of Large-Scale Brain Systems in Advanced Aging. Neuron.

[10] Lupien S.J., Maheu F., Tu M., Fiocco A., Schramek T.E. (2007). The Effects of Stress and Stress Hormones on Human Cognition: Implications for the Field of Brain and Cognition. Brain. Cogn..

[11] Arnsten A.F.T. (2009). Stress Signalling Pathways That Impair Prefrontal Cortex Structure and Function. Nat. Rev. Neurosci..

[12] Guergova S., Dufour A. (2011). Thermal Sensitivity in the Elderly: A Review. Ageing. Res. Rev..

[13] Yamashiro M., Nishimura Y., Mikami Y., Kouda K., Sakurai Y., Yoshioka I., Kinoshita T., Kojima D., Tajima F. (2020). Attenuation of Core Temperature Elevation and Interleukin-6 Excretion during Head-out Hot Water Immersion in Elderly People. J. Phys. Ther. Sci..

[14] Blatteis C.M. (2012). Age-Dependent Changes in Temperature Regulation—A Mini Review. Gerontology.

[15] O’Grady N.P., Barie P.S., Bartlett J.G., Bleck T., Carroll K., Kalil A.C., Linden P., Maki D.G., Nierman D., Pasculle W. (2008). Guidelines for Evaluation of New Fever in Critically Ill Adult Patients: 2008 Update from the American College of Critical Care Medicine and the Infectious Diseases Society of America. Crit. Care. Med..

[16] Liu K., Sun G., Li B., Jiang Q., Yang X., Li M., Li L., Qian S., Zhao L., Zhou Z. (2013). The Impact of Passive Hyperthermia on Human Attention Networks: An fMRI Study. Behav. Brain. Res..

[17] Sun G., Qian S., Jiang Q., Liu K., Li B., Li M., Zhao L., Zhou Z., von Deneen K.M., Liu Y. (2013). Hyperthermia-Induced Disruption of Functional Connectivity in the Human Brain Network. PLoS ONE.

[18] Brazaitis M., Eimantas N., Daniuseviciute L., Vitkauskiene A., Paulauskas H., Skurvydas A. (2015). Two Strategies for the Acute Response to Cold Exposure but One Strategy for the Response to Heat Stress. Int. J. Hyperth..

[19] Bayley P.J., Wixted J.T., Hopkins R.O., Squire L.R. (2008). Yes/No Recognition, Forced-Choice Recognition, and the Human Hippocampus. J. Cogn. Neurosci..

[20] Diamond A. (2013). Executive Functions. Annu. Rev. Psychol..

[21] Schlader Z.J., Gagnon D., Adams A., Rivas E., Cullum C.M., Crandall C.G. (2015). Cognitive and Perceptual Responses during Passive Heat Stress in Younger and Older Adults. Am. J. Physiol. Regul. Integr. Comp. Physiol..

[22] Baranauskiene N., Wang J., Eimantas N., Solianik R., Brazaitis M. (2023). Age-related Differences in the Neuromuscular Performance of Fatigue-provoking Exercise under Severe Whole-body Hyperthermia Conditions. Scand. J. Med. Sci. Sports.

[23] Leliavski A., Dumbell R., Ott V., Oster H. (2015). Adrenal Clocks and the Role of Adrenal Hormones in the Regulation of Circadian Physiology. J. Biol. Rhythm..

[24] Li S., Lu A., Li B., Wang Y. (2004). Circadian Rhythms on HyJpothalamic-Pituitary-Adrenal Axis Hormones and Cytokines of Collagen Induced Arthritis in Rats. J. Autoimmun..

[25] Moran D.S., Shitzer A., Pandolf K.B. (1998). A Physiological Strain Index to Evaluate Heat Stress. Am. J. Physiol..

[26] Solianik R., Skurvydas A., Mickevičienė D., Brazaitis M. (2014). Intermittent Whole-Body Cold Immersion Induces Similar Thermal Stress but Different Motor and Cognitive Responses between Males and Females. Cryobiology.

[27] Solianik R., Skurvydas A., Urboniene D., Eimantas N., Daniuseviciute L., Brazaitis M. (2015). Similar Cold Stress Induces Sex-Specific Neuroendocrine and Working Memory Responses. Cryo Lett..

[28] Solianik R., Brazaitis M., Skurvydas A. (2016). Sex-Related Differences in Attention and Memory. Medicina.

[29] Finkel D., Reynolds C.A., McArdle J.J., Gatz M., Pedersen N.L. (2003). Latent Growth Curve Analyses of Accelerating Decline in Cognitive Abilities in Late Adulthood. Dev. Psychol..

[30] Hedden T., Gabrieli J.D.E. (2004). Insights into the Ageing Mind: A View from Cognitive Neuroscience. Nat. Rev. Neurosci..

[31] Nyberg L., Lövdén M., Riklund K., Lindenberger U., Bäckman L. (2012). Memory Aging and Brain Maintenance. Trends Cogn. Sci..

[32] Salthouse T. (2012). Consequences of Age-Related Cognitive Declines. Annu. Rev. Psychol..

[33] De Souza-Talarico J.N., Marin M.-F., Sindi S., Lupien S.J. (2011). Effects of Stress Hormones on the Brain and Cognition: Evidence from Normal to Pathological Aging. Dement. Neuropsychol..

[34] Vedhara K., Hyde J., Gilchrist I.D., Tytherleigh M., Plummer S. (2000). Acute Stress, Memory, Attention and Cortisol. Psychoneuroendocrinology.

[35] Brazaitis M., Paulauskas H., Eimantas N., Daniuseviciute L., Volungevicius G., Skurvydas A. (2019). Motor Performance Is Preserved in Healthy Aged Adults Following Severe Whole-Body Hyperthermia. Int. J. Hyperth..

[36] Palve S.S., Palve S.B. (2018). Impact of Aging on Nerve Conduction Velocities and Late Responses in Healthy Individuals. J. Neurosci. Rural. Pract..

[37] Mather M. (2012). The Emotion Paradox in the Aging Brain. Ann. N. Y. Acad. Sci..

[38] Brazaitis M., Paulauskas H., Eimantas N., Obelieniene D., Baranauskiene N., Skurvydas A. (2017). Heat Transfer and Loss by Whole-Body Hyperthermia during Severe Lower-Body Heating Are Impaired in Healthy Older Men. Exp. Gerontol..

[39] Teichner W.H. (1958). Reaction Time in the Cold. J. Appl. Psychol..

[40] Kenny G.P., Yardley J., Brown C., Sigal R.J., Jay O. (2010). Heat Stress in Older Individuals and Patients with Common Chronic Diseases. CMAJ.

[41] Eimonte M., Paulauskas H., Daniuseviciute L., Eimantas N., Vitkauskiene A., Dauksaite G., Solianik R., Brazaitis M. (2021). Residual Effects of Short-Term Whole-Body Cold-Water Immersion on the Cytokine Profile, White Blood Cell Count, and Blood Markers of Stress. Int. J. Hyperther..

[42] Baker F.C., Siboza F., Fuller A. (2020). Temperature Regulation in Women: Effects of the Menstrual Cycle. Temperature.

[43] Neff L.M., Hoffmann M.E., Zeiss D.M., Lowry K., Edwards M., Rodriguez S.M., Wachsberg K.N., Kushner R., Landsberg L. (2016). Core Body Temperature Is Lower in Postmenopausal Women than Premenopausal Women: Potential Implications for Energy Metabolism and Midlife Weight Gain. Cardiovasc. Endocrinol..

